# Brain‐Wide Cellular‐Resolution Measurement of Antibody Therapeutic Biodistribution

**DOI:** 10.1002/alz.095666

**Published:** 2025-01-09

**Authors:** Chase Redd, Nathaniel Guanzon, Yessenia Gallegos, Jacob Weiss, Camilla Stampe Jensen, Sandra B. Vergo, Damian G. Wheeler

**Affiliations:** ^1^ Translucence Biosystems, Inc, Irvine, CA USA; ^2^ H. Lundbeck A/S, Valby Denmark; ^3^ Activity Signaling, LLC, San Diego, CA USA

## Abstract

**Background:**

To look deep inside tissues, traditional histological methods cut specimens into thin slices. Providing access to the intricate anatomy of intact organs, tissue clearing offers neuroscientists unbiased and complete views of brain anatomy and function. One area where these methods have particular utility is in the development of CNS therapeutics where they can be used to examine the regional distribution of the therapeutics in the brain as well as brain‐wide target engagement and phenotypic efficacy. We have developed a pipeline that provides unbiased and complete cellular resolution measurements of brain‐wide therapeutic biodistribution in pre‐clinical rodent brains.

**Methods:**

With our optimized iDISCO‐based clearing method and our Mesoscale Imaging System for ZEISS Lightsheet microscopes, we can image cellular‐resolution immunoreactivity across entire mouse brains in <20 min. Here we examined whether our technology can detect antibody therapeutics crossing the blood‐brain barrier (BBB). To do this, we took advantage of a bispecific antibody engineered to bind to the Alzheimer’s Disease target, BACE1, as well as the transferrin receptor (TfR1), which helps shuttle the antibody across the brain endothelium and into the brain parenchyma.

**Results:**

We demonstrated that our methods can detect IV‐dosed BACE1/TfR1 bispecific antibody throughout the brain. In mice dosed with a monospecific control antibody that does not bind TfR1, immunoreactivity was at background levels, similar to that seen in mice not dosed with any antibody. The bispecific therapeutic antibody was detected in the parenchyma and enriched in brain regions with high BACE1 expression, indicating that the antibody crosses the BBB and engages the target. However, there are high levels of bispecific antibody bound to TfR1 in the vasculature where is does not have access to drug targets. To quantify the effective parenchymal levels of the bispecific antibody, we have extended out AI‐powered quantification pipeline to segment the staining in the vasculature and measure only the therapeutically relevant bispecific antibody signal in the brain parenchyma.

**Conclusion:**

These data provide a clear demonstration of the utility of tissue clearing methods for quantitative brain‐wide monitoring of Alzheimer’s Disease therapeutic antibody biodistribution.

